# Apatinib manifests an unexpectedly favorable outcome in the management of axillary lymph node follicular dendritic cell sarcoma: a case report

**DOI:** 10.3389/fonc.2024.1388982

**Published:** 2024-06-19

**Authors:** Zhiying Feng, Zhonghai Du, Yan Liang, Juyue Zhou

**Affiliations:** ^1^ Graduate Institute, Shandong University of Traditional Chinese Medicine, Jinan, Shandong, China; ^2^ Department of Oncology, Weifang Hospital of Traditional Chinese Medicine, Weifang, Shandong, China

**Keywords:** apatinib, follicular dendritic cell sarcoma, axillary lymph node, treatment, case report

## Abstract

We present a case of follicular dendritic cell sarcoma in the axillary lymph node, which unexpectedly showed favorable outcomes after the application of apatinib. Follicular Dendritic Cell Sarcoma (FDCS) exhibits a rare incidence and an unclear pathogenic mechanism, contributing to the limited breakthroughs in its treatment to date within the medical field. The current mainstream therapeutic approaches include surgery, CHOP(cyclophosphamide, doxorubicin, vincristine, prednisone), ICE(ifosfamide, carboplatin, etoposide), ABVD(doxorubicin, bleomycin, vinblastine, dacarbazine), and immune checkpoint inhibitors. A 38-year-old male patient was admitted to the hospital due to a lump in the right axilla and underwent surgical treatment. Postoperative pathology confirmed the diagnosis of follicular dendritic cell sarcoma. Two months post-surgery, he faced a recurrence, prompting a subsequent surgical intervention complemented by tumor radiofrequency ablation. Despite these interventions, the treatment response was suboptimal. Subsequently, the patient was treated with the CHOP regimen, but after two cycles, he developed bone metastasis. Due to the patient's limited financial resources and refusal of immunotherapy, we switched to a regimen of gemcitabine and docetaxel, but the disease progressed again after two cycles. A one-cycle trial of albumin-bound paclitaxel yielded unsatisfactory results. Ultimately, the patient was treated with Apatinib, achieving a 10-month progression-free survival. Due to the patient's limited financial circumstances, we, in the absence of guideline recommendations and evidence from evidence-based medicine, achieved a 10-month progression-free survival (PFS) solely based on experiential use of the anti-angiogenic drug, Apatinib. The purpose of this case report is to provide additional therapeutic options for FDCS treatment and to pave the way for exploring the mechanism of action of Apatinib in FDCS.

## 1 Background

Follicular dendritic cell sarcoma (FDCS), initially reported by Monda et al. in 1986 (1), is a rare low-grade malignant tumor characterized by abnormal proliferation and differentiation of follicular dendritic cells (FDC) (2, 3). Some scholars also argue that it may exhibit features of moderate malignancy (4, 5). FDCS occurs in approximately 70% of cases within lymph nodes (5), with the most commonly affected sites being in the neck, axilla, and intra-abdominal lymph nodes (6). The incidence of local recurrence is 28.1%, and distant metastasis occurs in 27.2% of cases (5). Due to the low incidence of FDCS and a lack of prospective studies related to treatment, there is currently no standardized treatment protocol for this patient population. Apatinib is a selective tyrosine kinase inhibitor with a molecular weight of 493.178 g/mol and a molecular formula of C<sub>25</sub>H<sub>27</sub>N<sub>5</sub>O<sub>4</sub>S, derived from valatinib, targeting the inhibition of VEGFR-2 (6). Compared to anti-angiogenic drugs such as sorafenib, Apatinib exhibits a 10-fold higher affinity for VEGFR-2 (7, 8). Additionally, it possesses the capability to suppress other receptors, including c-kit, PDGFR-β, Ret, and c-src (9). The patient ultimately opted for Apatinib treatment, diverging from conventional optimal recommendations such as CHOP, ICE, ABVD, immune checkpoint inhibitors, etc. However, the achieved results were surprisingly favorable.

## 2 Case presentation

**Figure 1:**
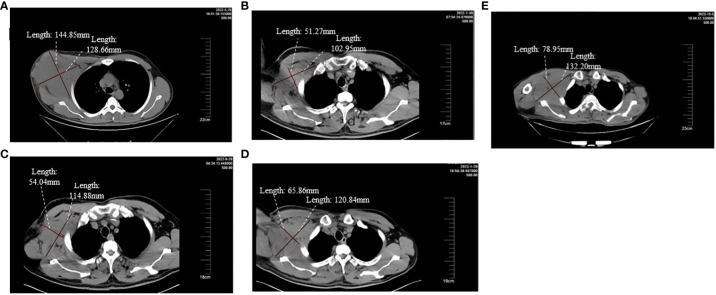
**(A-E)** respectively demonstrate the changes in the volume of the target lesion from late May 2022 before the second surgery to after using different treatment regimens.

**Figure 2:**
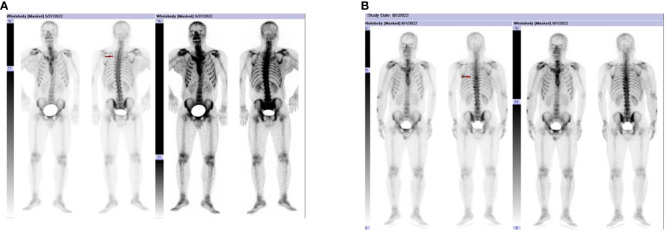
**(A, B)** show the patient developed bone metastases in early August 2022, prominently involving T6, T7, and T10. HE, 40 × magnification HE, 100 × magnification. HE, 200 × magnification HE, 100 × magnification.

**Figure 3:**
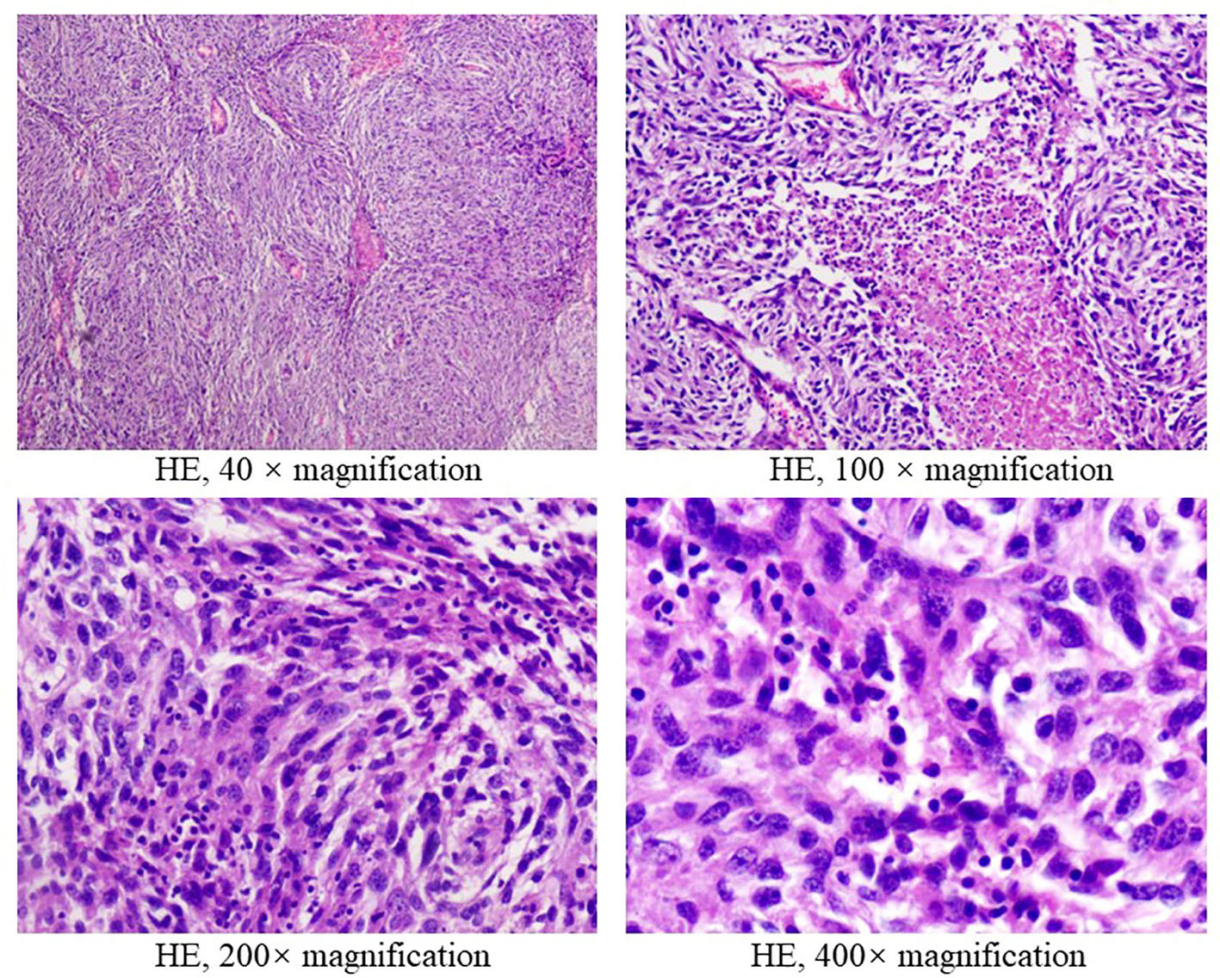
Hematoxylin & eosin stain of tumor biopsy specimens in 2022.

**Figure 4:**
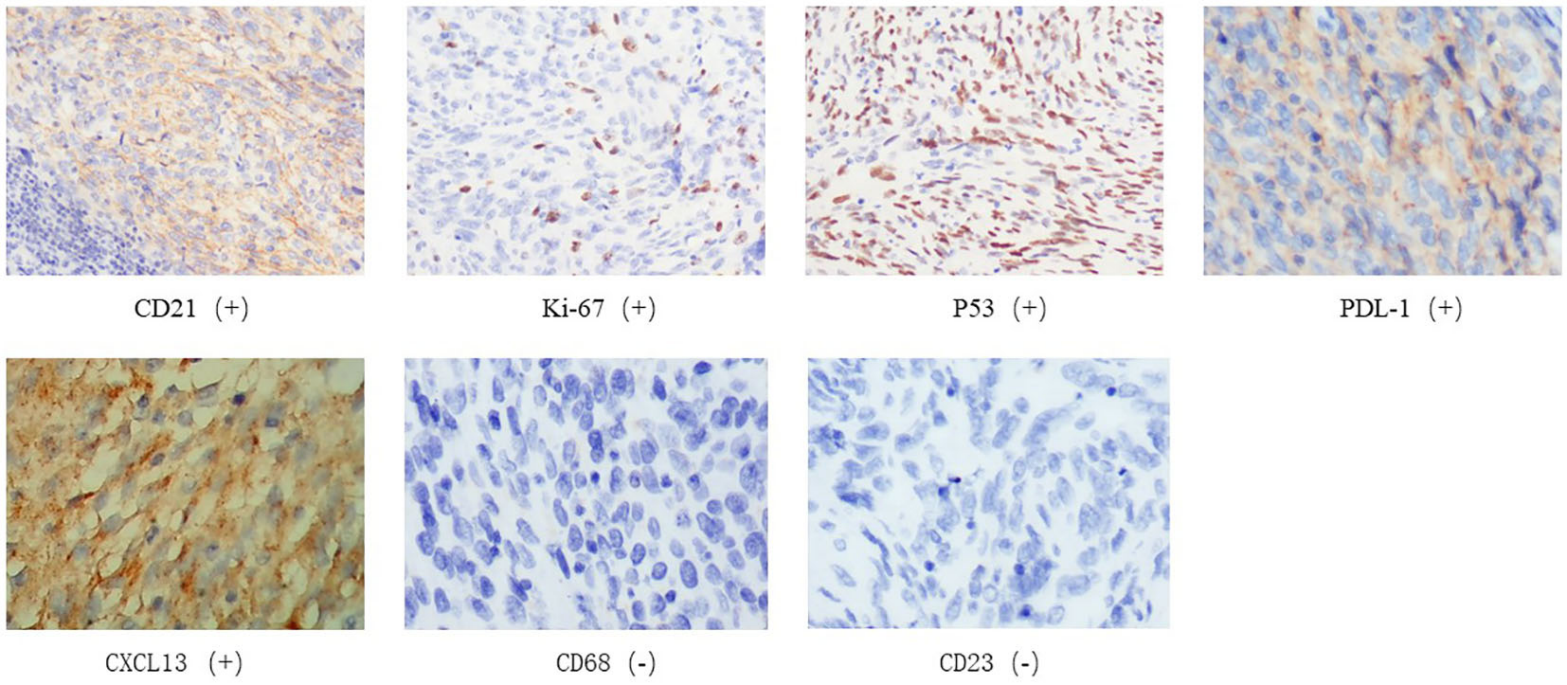
Immunohistochemistry of tumor biopsy specimens on May 30, 2022. CD21(+),Ki-67(+),P53(+),CD23(-),CD68(-),CXCL13(+),PDL-1(+).

A 38-year-old male underwent surgical treatment for a mass in the right axilla in March 2021. A total of three irregular, gray-white masses were excised, measuring 8cm×7cm×3.5cm in total. The postoperative pathology report indicates significant cellular atypia in specific regions, prominent mitotic activity, and observable coagulative necrosis, all consistent with a high-grade classification.CD21(+), CD23(-), CK(-), S-100(-), Melan-A(-), Ki-67(15%+), EMA(-), HMB45(-), CD68(-). The pathological diagnosis revealed follicular dendritic cell sarcoma in the (right axillary) lymph node. After the surgery, he refused any chemotherapy or radiation therapy due to poverty. He self-reported a progressive enlargement of the mass beneath the right axilla and above the right clavicle one month postoperatively. On May 23, 2022, he presented to our hospital due to the sudden onset of significant pain. ECT(emission computed tomography) and CT (computed tomography ) scans revealed no visceral or bone metastases. The maximum cross-sectional dimension of the target lesion measures approximately 117×130 mm (**Figures 1A**, **2A**). Subsequently, he underwent chest wall tumor resection surgery along with tumor radiofrequency ablation. Due to the indistinct boundary between the tumor and surrounding tissues, only palliative resection (R2 resection) can be performed. The ablation power is set at 50 watts for a duration of 1.5 minutes. After retracting the needle by 1cm, the power is adjusted to 40 watts for 2 minutes. The needle position is then readjusted for an additional 1.5 minutes of ablation. The postoperative pathology revealed: (Right axilla) Spindle cell proliferation with active growth, showing necrosis, nuclear division, and significant cellular atypia. (**Figure 3**) Immunohistochemistry demonstrated CD21 (+), CD23 (-), CD68 (-), CK (-), Ki67 (15%+), Melan-A (-), P53 (+), S-100 (-), MLH1 (+), MSH2 (+), MSH6 (+), PD-1 (-), PD-L1 (approximately 30% positive in tumor cells), PMS2 (+), CXCL13(+) (**Figure 4**). The pathological diagnosis was consistent with follicular dendritic cell sarcoma. Subsequently, the patient underwent CHOP chemotherapy regimen, which included intravenous cyclophosphamide (750mg/m2, day 1), intravenous doxorubicin hydrochloride (40–50mg/m2, day 1), intravenous vincristine sulfate (1.4mg/m2, day 1), and oral prednisone (100mg, days 1–5).

After completing two cycles of the prescribed regimen, the patient underwent a follow-up assessment In late July 2022. Compared to the examination at the end of May 2022, the maximum cross-sectional dimension of the target lesion is now approximately 103×51 mm. Considering the results of the ECT examination and the presence of bone metastasis, it indicates disease progression. (**Figures 1B**, **2B**). The patient declined immunotherapy due to economic reasons. Consequently, In early August 2022, he underwent a treatment regimen with gemcitabine hydrochloride (1.4g on days 1 and 8) in combination with docetaxel injection (65mg on days 1 and 8).

After two cycles of this regimen, the patient underwent a CT reassessment at the end of September 2022. (**Figure 1C**). Compared to the end of July 2022, the maximum cross-sectional dimension of the target lesion is approximately 115×54mm. There were multiple low-density lesions observed in the vertebral bodies of the thoracic and lumbar spine, the fifth rib on the left side, the iliac bones on both sides, and the pubic bone on the right side, indicating disease progression.

Given the patient's condition involving bone metastasis, we initiated zoledronic acid for anti-metastatic bone treatment. The patient declined the recommended palliative radiation to local site or to bony metastatic lesions. In early October 2022, the patient received a regimen involving albumin-bound paclitaxel (200mg on day 1, 300mg on day 5). After one treatment cycle, the patient's condition showed improvement. At the end of November 2022 during the hospital follow-up examination, the patient declined a CT scan due to economic reasons. However, on physical examination, a noticeable increase in the size of the mass in the right axilla, exceeding 20% of the sum of diameters, was observed. Based on the RECIST 1.1 evaluation criteria, the patient's condition was assessed as deteriorating. The patient declined to continue the treatment with the ICE regimen. Subsequently, in the absence of specific cancer treatment guidelines and evidence from evidence-based medicine, considering the limited financial resources of the patient and the availability of a drug donation policy for purchasing Apatinib in China, compared to other agents with similar activity such as sorafenib, its advantages lie in its lower cost, higher affinity for VEGFR-2, and the characteristic of inhibiting PDGFR-β, a receptor closely associated with FDC. Solely based on clinical experience, we recommend the use of the anti-angiogenic drug Apatinib (250mg qd) for treatment. The patient agreed to this plan, and it was initiated on November 29, 2022. Throughout the course of treatment, the patient's condition remained stable with no significant adverse events observed, and overall health status was satisfactory.

The patient underwent an evaluation examination at the end of January 2023. Compared to the examination at the end of September 2022, the maximum cross-sectional size of the lesion was approximately 120×65mm. According to the RECIST 1.1 assessment criteria, the patient was classified as having stable disease (SD) (**Figure 1D**)

**Figure 5:**
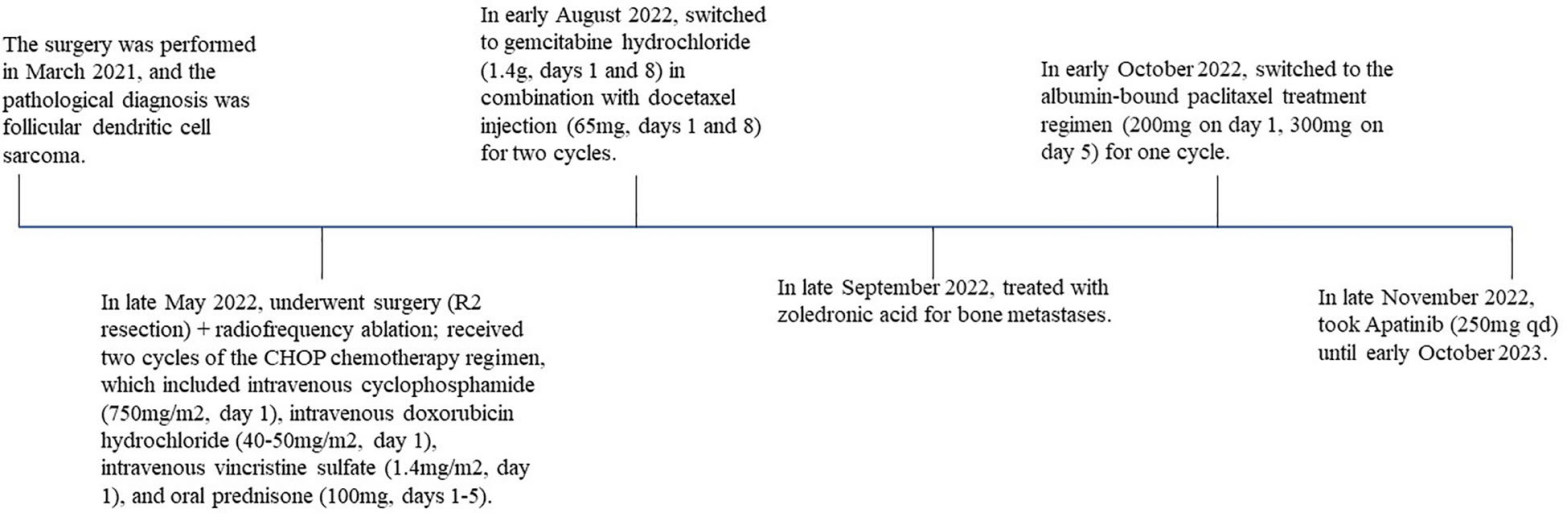
The patient’s treatment timeline.

Following this, the patient consistently maintained Apatinib treatment. However, it was not until early October 2023, when he was admitted for treatment after the rupture of a swelling below the right axilla, that a CT examination could be conducted. Compared to late January 2023, the maximum cross-sectional dimension of the target lesion is approximately 132×79 mm. Additionally, there are multiple metastatic lesions in the ribs, vertebral bodies of the thoracic and lumbar spine, appendicular bones, bilateral iliac bones, sacrum, right pubic branch, and right ischium. The assessment indicates disease progression (**Figure 1E**). Up to this point, the patient has been treated with Apatinib, achieving a PFS of 10 months. The entire treatment process is illustrated in the diagram (**Figure 5**).

## 3 Discussion and conclusions

To the best of our knowledge, this is the first report demonstrating a favorable response and prolonged PFS in metastatic FDCS treated with the single-agent anti-angiogenic drug, Apatinib, as a fourth-line treatment. FDCS patients span a broad age range, with a higher incidence in adults compared to children, and no significant gender difference in disease prevalence (10). However, the incidence appears to be higher in Asia (11). Characterized by slow growth, distinct borders, and mild pain, FDCS typically exhibits a texture ranging from soft to slightly firm, possibly involving regions of necrosis and bleeding (12). The unclear pathogenesis presents challenges in the diagnosis and treatment of this condition. FDCS tumor cells typically show positive results for CD21, CD23, and CD35, but lack specificity for markers such as S-100 and CD68 (13). The patient’s age, tumor size, lymphoplasmacytic infiltration, tumor cell mitotic count, necrosis, and lymph node metastasis are closely associated with the biological behavior and prognosis of FDCS (11).

Due to the low incidence of FDCS and a lack of prospective studies related to treatment, there is currently no standardized treatment protocol for this patient population. For patients with localized disease, optimal treatment involves surgical resection, with or without adjuvant chemotherapy and/or radiotherapy (14). However, studies have shown recurrence rates of 40%, 56%, and 20% for surgery alone, chemoradiotherapy, and combined treatment, respectively (15). Therefore, we can infer that adjuvant radiotherapy after surgery may contribute to improving patient prognosis. For patients who are not candidates for surgical intervention or have metastatic disease, the most commonly used regimen at present is CHOP (cyclophosphamide, doxorubicin, vincristine, prednisone) (16). In addition, ICE (ifosfamide, carboplatin, etoposide), gemcitabine plus paclitaxel, and ABVD (doxorubicin, bleomycin, vinblastine, dacarbazine) regimens are also frequently employed in clinical practice (2). Most FDCS patients opt for conventional treatment modalities, including surgery, radiotherapy, chemotherapy, etc., while a small minority opt for alternative treatment approaches. By searching literature published on PubMed, a comparison was made among patients opting not solely for traditional treatment approaches (2, 13, 17–21). (**Supplementary Table 1**) This revealed a significant extension in PFS among patients opting for immunotherapy. For patients exhibiting high PD-1 or PD-L1 expression, the administration of immune checkpoint inhibitors has proven to be crucial in enhancing patient PFS (2, 13, 20). His immunohistochemistry results indicated a PD-L1 positivity rate of 30%, according to the current consensus on diagnostic and therapeutic approaches, this patient meets the criteria for receiving immune checkpoint inhibitors. However, due to economic factors, the patient refuses immunotherapy. Due to clinical trials demonstrating limited survival benefits when anti-angiogenic agents are administered as monotherapy (22), coupled with the rarity of FDCS and its low incidence rate, there are currently almost no cases of using anti-angiogenic agents alone in the treatment of FDCS. Currently, only a few anti-angiogenic drugs are available for the treatment of various types of cancers. Apatinib is an orally bioavailable small-molecule agent that hinders angiogenesis by suppressing VEGF-mediated migration and proliferation of endothelial cells, thereby impeding the formation of new blood vessels in tumor tissues (23), and it exhibits anti-tumor effects in various types of tumors (24). Apatinib has demonstrated definite efficacy against gastric cancer, liver cancer, breast cancer (25), and its cost is relatively low. Therefore, with the patient’s and their family’s consent, in the absence of recommended cancer treatment guidelines and supporting evidence from evidence-based medicine, we have chosen to use Apatinib based solely on experiential prescription until now. Krautler et al. found that the precursor of FDC is present in platelet-derived growth factor receptor β (PDGFR-β) cells within the lymphatic and non-lymphatic vascular systems. Thus, FDC originate from ubiquitous vascular PDGFR-β precursors surrounding the vessels (26). We speculate that this may be one of the reasons for the favorable effects achieved with the use of Apatinib as a monotherapy for FDCS.

Our case report provides clinical evidence of the effectiveness and manageable safety of the use of the anti-angiogenic drug, Apatinib, as a subsequent treatment in a patient with postoperative recurrence and bone metastasis of FDCS. However, the patient, due to financial constraints, declined regular follow-up and imaging examinations as per the treatment plan, resulting in incomplete clinical assessment evidence, which is also a limitation of this case report. Up to the present, the patient has achieved a PFS of 10 months. Subsequently, the patient discontinued the use of Apatinib due to disease progression and the occurrence of oral ulcers. We will conduct regular follow-up observations to assess the patient’s overall survival (OS). In future research endeavors, it remains crucial to enhance the sample size and extend the observation duration to further substantiate its clinical applicability.

## Data availability statement

The original contributions presented in the study are included in the article/**Supplementary material**. Further inquiries can be directed to the corresponding author.

## Ethics statement

Written informed consent was obtained from the individual(s) for the publication of any potentially identifiable images or data included in this article.

## Author contributions

ZF: Investigation, Writing – original draft, Writing – review & editing. ZD: Investigation, Supervision, Writing – review & editing. YL: Supervision, Writing – review & editing. JZ: Supervision, Writing – review & editing.
