# Supplementary material for: Apatinib manifests an unexpectedly favorable outcome in the management of axillary lymph node follicular dendritic cell sarcoma: a case report
Source: Front Oncol. 2024 Jun 19;14:1388982. doi: 10.3389/fonc.2024.1388982 (PMC11219944; doi:10.3389/fonc.2024.1388982)
Supplement: Supplementary file 1 [file Table_1.docx]

The summary of FDCS patients who received treatment options other than chemotherapy and radiotherapy.

| Publication year | Country | Age**/**sex | Location | Treatment | Metastasis | PFS |
| --- | --- | --- | --- | --- | --- | --- |
| 2007 | Egypt | 49**/**M | submandibular region | radiotherapy**/**ifosfamide+dox-orubicin**/**gemcitabine+cisplatin+imatinib | liver, lung | 8 months |
| 2017 | Japan | 42**/**F | pancreas | S-1+Gemcitabine**/**DXR**/**IFM**/**CHOP**/**ESHAP**/**PTX**/**bendamustine | liver, lymph node, peritoneum | No data |
| 2020 | USA | 26**/**M | lung | CHOP**/**Gemcitabine+Taxotere**/**pembrolizumab+radiotherapy**/**  pazopanib | right external iliac, inguinal nodes | 9 months |
| 2020 | USA | 50**/**F | Retroperitoneum | surgery**/**VAdrC**/**radiotherapy+Nivo+Ipi | liver | 9 months |
| 2020 | USA | 40**/**F | pelvic appendage | surgery**/**Nivo+Ipi | peritoneum | 7 months |
| 2021 | China | 57**/**M | rectovesical pouch | surgery**/**CHOP**/**ABVD/sintilimab+lenvatinib, | mesentery, pelvic cavity | 7 months |
| 2022 | China | 51/M | neck | CHOP/vindesine+liposomal doxorubicin+ifosfamide+  methylprednisolone+radiotherapy/pembrolizumab | neck lymph node | 24 months |
| 2023 | China | 67/F | spleen | surgery**/**AI+sintilimab+radiotherapy | peritoneum | 17 months |
| 2024 | China | 59/M | gallbladder | Sintilimab+anlotinib+paclitaxel albumin | liver | 30months |
| M Male, F Female, The treatment plans are arranged in chronological order. | | | | |  |  |
